# Exome sequencing identifies a disease variant of the mitochondrial ATP‐Mg/Pi carrier SLC25A25 in two families with kidney stones

**DOI:** 10.1002/mgg3.1749

**Published:** 2021-08-04

**Authors:** M. Reza Jabalameli, Fiona M. Fitzpatrick, Roberto Colombo, Sarah A. Howles, Gary Leggatt, Valerie Walker, Akira Wiberg, Edmund R. S. Kunji, Sarah Ennis

**Affiliations:** ^1^ Department of Human Genetics and Genomic Medicine University of Southampton Southampton UK; ^2^ Medical Research Council Mitochondrial Biology Unit University of Cambridge Cambridge UK; ^3^ Faculty of Medicine ‘Agostino Gemelli’ Catholic University of the Sacred Heart Rome Italy; ^4^ Center for the Study of Rare Inherited Diseases Niguarda Ca´Granda Metropolitan Hospital Milan Italy; ^5^ Nuffield Department of Surgical Sciences University of Oxford Oxford UK; ^6^ Wessex Kidney Centre Queen Alexandra Hospital Portsmouth UK; ^7^ Department of Clinical Biochemistry University Hospital Southampton Southampton UK; ^8^ Nuffield Department of Orthopaedics, Rheumatology and Musculoskeletal Sciences University of Oxford Oxford UK; ^9^ Academic Endocrine Unit, Radcliffe Department of Medicine University of Oxford Oxford UK; ^10^ Present address: Department of Genetics Albert Einstein College of Medicine Bronx New York 10461 USA

**Keywords:** calcium kidney stones, calcium signaling, mitochondrial adenine nucleotide metastasis, mitochondrial transporter, purinergic signaling

## Abstract

**Background:**

Calcium kidney stones are common and recurrences are often not preventable by available empiric remedies. Their etiology is multifactorial and polygenic, and an increasing number of genes are implicated. Their identification will enable improved management.

**Methods:**

DNA from three stone‐formers in a Southampton family (UK) and two from an Italian family were analyzed independently by whole exome sequencing and selected variants were genotyped across all available members of both pedigrees. A disease variant of *SLC25A25* (OMIM 608745), encoding the mitochondrial ATP‐Mg/Pi carrier 3 (APC3) was identified, and analyzed structurally and functionally with respect to its calcium‐regulated transport activity.

**Results:**

All five patients had a heterozygous dominant *SLC25A25* variant (rs140777921; GRCh37.p13: chr 9 130868670 G>C; p.Gln349His; Reference Sequence NM_001006641.3). Non‐stone formers also carried the variant indicating incomplete penetrance. Modeling suggests that the variant lacks a conserved polar interaction, which may cause structural instability. Calcium‐regulated ATP transport was reduced to ~20% of the wild type, showing a large reduction in function.

**Conclusion:**

The transporter is important in regulating mitochondrial ATP production. This rare variant may increase urine lithogenicity through impaired provision of ATP for solute transport processes in the kidney, and/or for purinergic signaling. Variants found in other genes may compound this abnormality.

## Graphical abstract

Independently, whole exome sequencing to look for monogenic mutations in two families with autosomally inherited calcium kidney stone disease, identified a disease variant of *SLC25A25*, encoding the mitochondrial ATP‐Mg/Pi carrier 3 (APC3). Modeling suggests that the variant lacks a conserved polar interaction, which may cause structural instability. Calcium‐regulated ATP transport was reduced to ~20% of the wild type, showing a large reduction in function. This rare variant may increase urine lithogenicity through impaired provision of ATP for solute transport processes in the kidney, and/or for purinergic signaling.

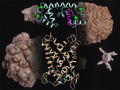

## 1 INTRODUCTION

Renal stones are aggregates of inorganic and/or organic crystals formed within the kidneys. They are common worldwide with recent estimated lifetime prevalence in men of around 10%. They often recur and this may impact significantly on the lives of stone formers. More than 75% are calcium oxalate stones, often mixed with calcium phosphate. Most of these are idiopathic. Many factors contribute to their formation, notably an increased concentration of minerals due to low fluid intake or dietary excesses, hormonal imbalance, and decreased protection by endogenous inhibitory agents in urine (Coe et al., 2005; Moe, 2006; Prochaska et al., 2016; Walker et al., 2013). These factors are variable and change throughout life. However, there is also an underlying genetic component, with heritability estimated at about 50%. Around 20% of stone formers have a positive family history of stones compared with 6% among non‐stone formers (Coe et al., 2005; Curhan et al., 1997; Gambaro et al., 2016; Goldfarb et al., 2019; Walker et al., 2013). Well‐established monogenic causes of stone disease are being recognized increasingly in individuals referred to specialist clinics because of significant stone problems without a previous genetic diagnosis (Braun et al., 2016; Daga et al., 2018; Gambaro et al., 2016; Halbritter et al., 2015; Sayer, 2017; Vezzoli et al., 2011). However, in the majority of stone formers, genetic susceptibility is polygenic with co‐inheritance of multiple small effect gene variants. GWAS studies have identified common polymorphisms (MAF > 1%) in 31 genes associated with stone formation, most with modest effects (OR < 1.5; Howles & Thakker, 2020; Moe, 2006; Palsson et al., 2019). In other complex polygenic disorders such as diabetes mellitus, common polymorphisms do not fully account for genetic susceptibility. Rare/low‐frequency variants are likely to contribute to the missing genetic component (Pang et al., 2021). Current interventions to prevent stone recurrence are still largely empiric and often ineffective (Gambaro et al., 2016). Progress with targeted treatment and prevention demands a better understanding of the underlying genetic factors.

Two decades ago, we undertook a small study in Southampton to look for variants in genes relevant to stones in 14 kindreds in which at least three first‐degree relatives had had calcium stones (Walker & Griffin, 2015). Family members had biochemical investigation for stone risk factors. Mouthwash DNA was analyzed using microsatellites to investigate the segregation of four genes with stone formation (*Vitamin D Receptor (VDR*; OMIM 601769*)*, *Calcium Sensing Receptor (CASR*; OMIM 601199*)*, *Thiazide*‐*sensitive Sodium Chloride Transporter (SLC12A3*; OMIM 600968) and *Sodium Phosphate Transporter 2a (SLC34A1* OMIM 182309). The results were negative. Many other studies have looked for polymorphisms in candidate genes known to influence urine composition which might increase the risk for stones. No common clear leaders have emerged (Palsson et al., 2019; Sayer, 2017; Vezzoli et al., 2011; Walker, 2019).

With whole exome sequencing (WES) and genome‐wide association studies (GWAS), there is the potential to extend the search to the wide range of intracellular proteins that underpin urine production (Howles et al., 2019; Palsson et al., 2019; Tanikawa et al., 2019; Walker, 2019). In order to look further for gene variants responsible for increasing risk for kidney stones in these families, we have now analyzed blood stored from the study by WES.

A heterozygous dominant rare variant in the gene *SLC25A25* (*Solute Carrier Family 25 member 25)* was identified in stone formers in one Southampton kindred. This encodes the mitochondrial ATP‐Mg/Pi carrier 3, APC3 (aliases SCaMC2 [small calcium‐binding mitochondrial carrier 2], and SLC25A25 [solute carrier family 25 member 25] [del Arco & Satrústegui, 2004; Fiermonte et al., 2004]). The same variant was identified independently in an Italian pedigree, and segregated well with stone formation.

## 2 PATIENTS AND METHODS

### 2.1 The families

**FIGURE 1:**
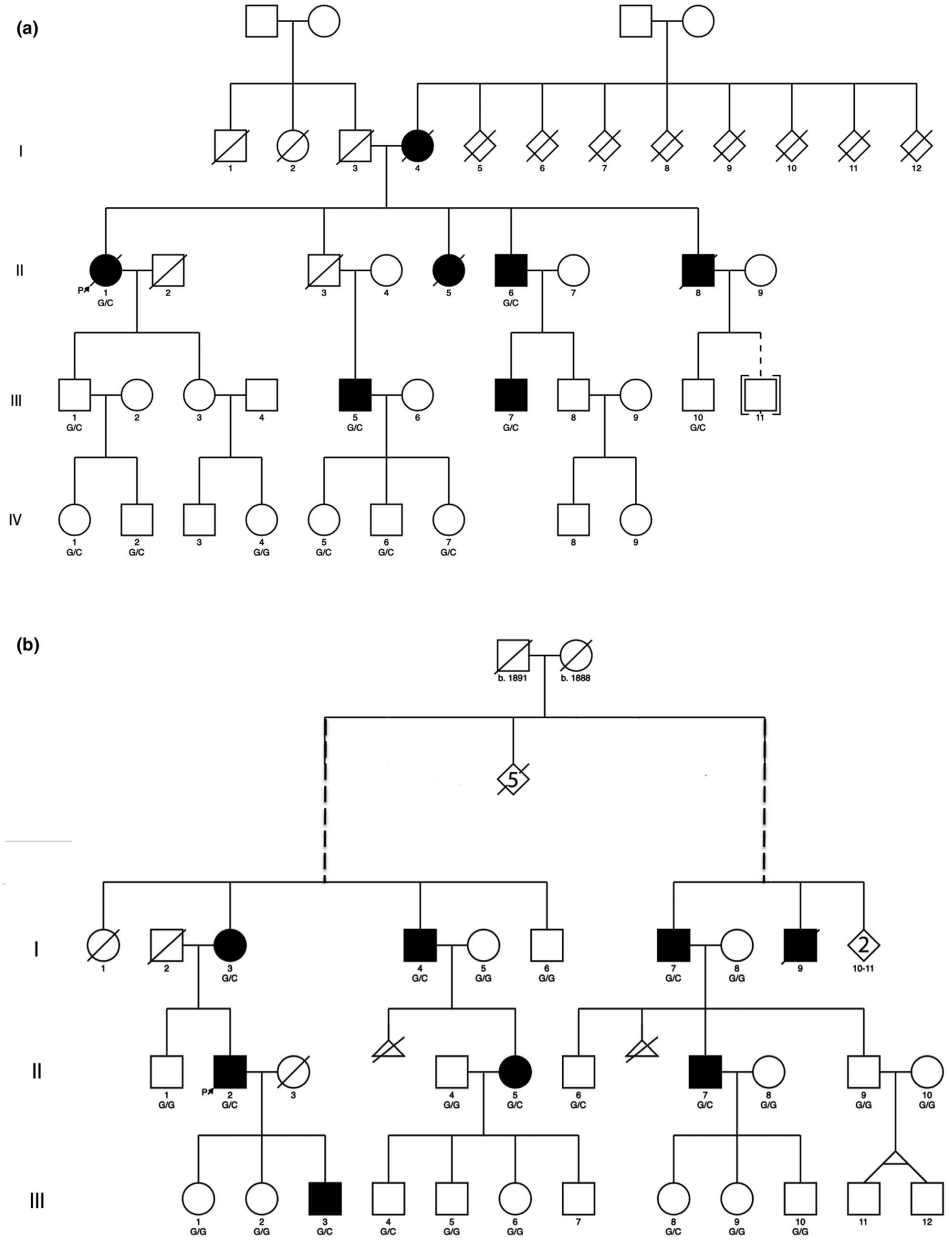
Pedigrees of the two families showing apparent autosomal dominant inheritance of stones. (a) UK family: DNA from individuals II‐1, III‐5, and III‐7 were analyzed by WES; segregation of the variant in the family was by KASPar genotyping. (b) Italian kindred: The left and the right branch of the pedigree trace their ancestry to a common family founder, indicated by the dashed lines. DNA from individuals II‐2, III‐3 were analyzed by WES and the variant confirmed by Sanger sequencing. Segregation of the variant was by restriction analysis; Solid symbols: stone formers; Δ‐miscarriages

The Southampton family was initially recruited to our study in 1998. Seven individuals spanning three generations were stone formers (3 deceased; Figure 1a). The propositus (II‐1), a 79‐year‐old patient of the Southampton stone clinic had her first stone aged 44 years and another, containing calcium, oxalate, and phosphate, at 78 years. Her mother (I‐4) had a nephrectomy for stones aged 39 years. Four other family members had renal stones aged 18–64 years. A further male patient (II‐6) is not known to have had kidney stones but formed 2 cm bladder calculus on an indwelling catheter for Parkinson's disease. Twelve adults ≥18 years of age provided blood and urine for biochemical investigations and blood and mouth wash samples for DNA analysis. None of the family had primary hyperparathyroidism or, with the exception of patient II‐6, another underlying medical disorder associated with urinary tract stones. At the time of consenting to the follow‐up study in 2016, the participants were asked to complete a short questionnaire (Document S1) asking whether they had formed more stones, had chronic illnesses, repeat medications and whether any of their children or grandchildren had had kidney stones. In addition, their records held on the hospital computer system were reviewed for relevant clinical and biochemical data. DNA extracted from blood stored at −20℃ from stone formers II‐1, III‐5, and III‐7 was analyzed by WES. Segregation of selected rare variants with stones was investigated by genotyping DNA from all 12 participants.

The propositus in the family from Southern Italy sought counselling aged 51 years because of recurrent stones, having had three removed from 32 years of age. He was physically very active and otherwise healthy, with no history of hypertension or diabetes and was not taking medications. His mother (I‐3 in Figure 1b), maternal uncle (I‐4, 76 years), and youngest son (III‐3, 21 years) had one or more kidney stones. From family members and contacts with general practitioners another five related subjects were identified spanning three generations, four still alive in 2016 (I‐7, II‐5, II‐7, and III‐8) and one deceased (I‐9). Through consultation of parish and civil registers of births and marriages the family was found to have two branches with the common ancestors born in 1888 and 1891. All participants provided a blood sample or buccal swab for genotyping. Samples from II‐2 and III‐3 were analyzed by WES and the *SLC25A25* mutation was confirmed by Sanger sequencing. Candidate variant alleles were genotyped in the pedigree using endonuclease restriction analysis.

### 2.2 Biochemical investigations in Southampton 1998: derived values

Using creatinine clearance as a surrogate for inulin clearance and glomerular filtration rate, and 60% of albumin‐corrected serum calcium for ultrafilterable calcium, approximate values for (a) *fasting tubular reabsorption of Ca* was estimated from the paired fasting blood and random urine samples: 1‐Ca clearance/creatinine clearance × 100%; [=1−urine Ca × plasma creatinine/urine creatinine × plasma ultrafilterable Ca × 100%], and (b) *percent of Ca reabsorbed over 24 hr* = Ca reabsorbed/Ca filtered over 24 hr × 100%, where filtered Ca (mmol/24 hr) = ultrafilterable Ca x creatinine clearance (L per 24 hr) and reabsorbed Ca = filtered‐24 hr urine Ca (mmol/24 hr). To assess tubular phosphate reabsorption, *t* *he renal threshold phosphate concentration (TmPO4*/*GFR)* was estimated from paired fasting blood and urine samples using a nomogram (Walker & Griffin, 2015; Walker et al., 2013).

### 2.3 Gene analyses

#### 2.3.1 Exome sequencing

*In Southampton*: Extracted DNA was assessed for quality and analyzed by WES. Library capture was performed using the Agilent SureSelect All Exon V.5 and sequenced using Illumina HiSeq platform. Raw sequence data were analyzed using a local pipeline for sequence alignment to human genome hg19/GRCh 37. To maximize sensitivity, variants for individual samples were called contemporaneously in Samtools v1.3.2 and GATK (v3.6) HaplotypeCaller. The resultant merged VCF files were annotated using ANNOVAR (2015 Dec 14 release). The sequences were first cross‐referenced against a panel of 366 genes relevant to stones which we extracted from published data (Table S1). Variants were then excluded if: they were common (minor allele frequency (MAF) >0.02 in the 1000 Genome project); present in homopolymer tracts or repeat regions; had read depth <10; were located in highly mutable genes; had a strand‐, or base quality bias and; were not identified in all three stone formers. This analysis was followed by more aggressive filtering of data outside the 366 gene‐panel with exclusion of synonymous, non‐frameshift insertion or splicing or ncRNA splicing variants, or those with frequency of >2% in the in‐house Southampton database of around 600 individual exomes. All remaining variants were then prioritized through multidisciplinary team discussion according to rarity (EXAC, 1000 Genomes), predicted pathogenicity (SIFT, site conservation [Gerp++, Phylop scores]) and likely clinical relevance.

*In Italy*: WES was undertaken by the Centre for Applied Genomics (TCAG; Toronto, Ontario, Canada). Paired end sequencing of DNA fragments (average 200 bp) was carried out using the HiSeq 2500 platform (Illumina Inc.,). Sequence reads were trimmed using Trimmomatic (Bolger et al., 2014) and aligned to the reference human genome sequence (hg19/GRCh 37). Single nucleotide variants and indels were called using GATK haplotype caller v. 3.2.2. (DePristo et al., 2011). Variants were filtered to identify rare polymorphisms using publicly available databases (1,000 Genomes; Exome Aggregation Consortium [ExAC] browser, NHLBI Exome Sequencing Project [ESP], and dbSNP).

#### 2.3.2 Genotyping

In Southampton, selected variants were analyzed for segregation across all available family members using KASPar genotying (KASP TM; LGC, Hoddesdon). In Italy, the c.1047G>C variant (transcript reference sequence NM_001006641.3) was confirmed in the proband (II‐2) and his son (III‐3) by bidirectional Sanger sequencing of the PCR‐amplified *SLC25A25* primers 3′‐CTGCTCCTGTTGTGCAGGT‐5′ (forward; Tm 60℃) and 3′‐TGCTGGGAGGAGGTTTCTAA‐5′ (reverse; Tm 59.8℃). Other family members were genotyped by restriction analysis of the 227‐bp PCR amplicon with *Bsr*I or *Rsa*I (New England Biolabs) whose restriction sites are abolished by the c.1047G>C transversion.

### 2.4 Computational modeling of APC3b

Models of the APC3 isoform b, APC3b, were generated in MODELLER (Webb & Sali, 2014), using the bovine mitochondrial ADP/ATP carrier (adenine nucleotide translocase) AAC1 (PDB: 1okc) and the fungal ADP/ATP carrier structures (PDB:4c9g, 4c9h, 4c9j, 4c9q) as templates (Pebay‐Peyroula et al., 2003; Ruprecht et al., 2014). The structure of the calcium‐regulatory domain was taken from PDB:4zcu (Harborne et al., 2015). Models were visualized and the most likely rotamer for the histidine substitution at position 349 was selected in PyMOL Molecular Graphics System, Version 1.8 (Schrödinger LLC).

### 2.5 Expression, purification, and functional characterization of wild‐type and mutant APC3b

The human wild‐type APC3b and the disease variant were expressed in yeast mitochondria, purified, analyzed by thermal stability assays and reconstituted, as described previously (Hofherr et al., 2018). Briefly, purified protein (~60 μg) was reconstituted into liposomes containing L‐α‐phosphatidylcholine (Avanti Polar Lipids) and tetraoleoyl cardiolipin (Avanti Polar Lipids) in a 20:1 (w/w) ratio. The detergent pentaethylene glycol monodecyl ether was added to a final concentration of 1.6% (v/v) to solubilize the lipids, and the detergent was removed by multiple additions of SM‐2 bio‐beads (Bio‐Rad) in the presence of 1 mM ATP‐Mg. The external substrate was removed using a PD10 desalting column (GE Healthcare). Transport rates were determined by measuring the uptake of 2 μM [^14^C]‐ATP with or without the addition of 1 mM CaCl_2_ over a 15‐min time‐course. Uptake curves were fitted with a One‐Phase Association model (GraphPad, Prism).

### 2.6 Protein analysis

Proteins were separated using precast 4%–12% TruPAGE^™^ SDS–PAGE gels (Merck KGaA), loaded with 5 µg of protein at 3:1 mix of sample to loading buffer and stained using InstantBlue^™^ Coomassie (Merck KGaA). Protein concentration was determined by measuring the absorbance at 280 nm using a NanoDrop ND‐1000 Spectrophotometer (NanoDropTechnologies).

### 2.7 UK Biobank search for the selected rare gene variants

UK Biobank is a prospective cohort study to investigate risk factors for the major diseases of middle and old age. Between 2006 and 2010 the study recruited around 500,000 men and women aged 40–69 years who have had whole genome genotyping undertaken and allowed linkage of their data with their medical records (Collins, 2012). A genome‐wide association study of renal stone disease has recently been undertaken in UK Biobank from 6,536 stone formers (66.7% men) and 388,508 controls (45.5% men) of white British ancestry, mean ages (SD) 67.9 (7.63) years and 66.8 (8.01) years, respectively (Howles et al., 2019).

We sought to compare the population frequencies of the six variants prioritized from the Southampton WES data (*SLC25A25*, *VPS16*, *PLA2R1*, *MAP3K5*, *PKP4*, and *HAVCR1*) in the 6,536 Biobank stone formers and 388,508 non‐stone formers from the GWAS. Genotype data were available for five out of six of these variants (all except the *HAVCR1* variant, which was not present on the UK Biobank Axiom array). Minor allele frequencies were compared for stone formers versus non‐stone formers using PLINK, a tool for GWAS and population‐based linkage analysis (Purcell et al., 2007), and a chi‐squared test was performed. A Bonferroni correction was applied to account for multiple testing, with the significance threshold set at *p* < .01 (0.05/5).

## 3 RESULTS

### 3.1 Follow‐up of Southampton patients

From the questionnaire in 2016, no new stone formers were identified in the family and none of the children of generation IV had had stones. From the participants and hospital records, three family members (I‐4, II‐3, and III‐5) had gallstones, one had developed diabetes mellitus, and five had hypertension. None had chronic muscle problems, but on separate courses of two statins for polygenic hyperlipidemia, one participant had muscle pains. There were no results available for creatine kinase during these events (Table S2).

### 3.2 Biochemical assessment in 1998: Southampton family

The frequent biochemical risk factor identified in 1998 was a decrease in renal phosphate reabsorption, (“hyperphosphaturia”) in 6 of 12 participants (renal threshold phosphate concentration, TmPO4/GFR <0.80 mmol/L; Table S3). Only one individual (IV‐6) had an increased 24 hr urine calcium excretion, and across the pedigree calcium was reabsorbed well from the kidneys, exceeding 97% in all but two of the 12 participants (II‐6 and IV‐6) over 24 hr, and in one (II‐6) when fasting. Patient II‐6 had an indwelling catheter and bladder stone.

### 3.3 Identification of a rare variant in *SLC25A25* and other rare variants among the stone formers

**FIGURE 2:**
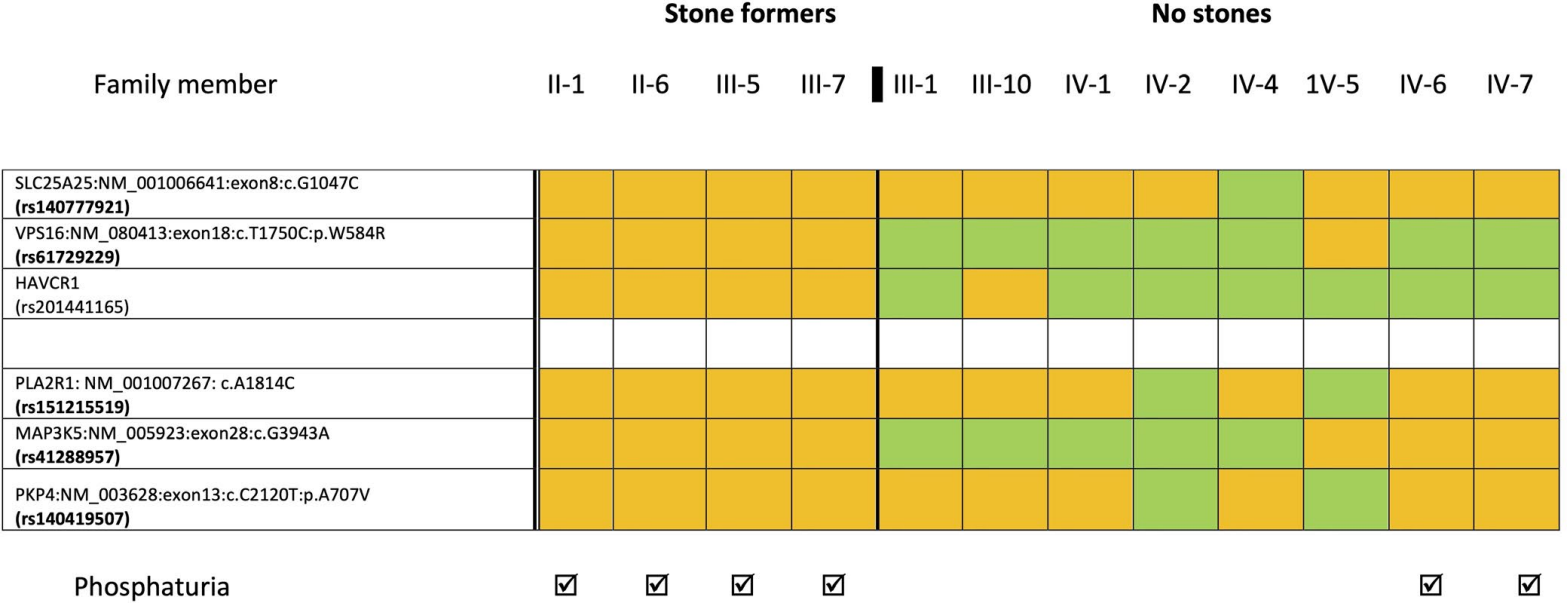
Segregation of gene variants & phosphaturia in the Southampton kindred. Rare variants in six genes identified by WES in stone formers II‐1, III‐5, and III‐7 were selected for their possible relevance to stone formation in this kindred. These variants were then analyzed for segregation across all 12 available family members using KASPar genotyping (KASP TM; LGC, Hoddesdon, Herts, UK). Orange indicates heterozygosity for the rare allele; green indicates the common allele

Initial filtering of whole exome variants in Southampton identified two variants that fulfilled all selection criteria. One (rs41288957), in *MAP3K5* (*mitogen activated kinase 5*, *ASK1*, OMIM 602448) had predicted low pathogenicity (SIFT 0.36, Polyphen: 0.002). The other (rs140777921) was in *SLC25A25* (dbSNP: chr9: 130868670G>C, (GRCh37.p13). This gene had been included in the candidate gene list because its expression (mRNA) in mouse kidney changed significantly with glyoxylate‐induced calcium oxalate crystallization (Okada et al., 2009). The second round of filtering identified rare, predicted deleterious, variants in four genes expressed in kidneys, but with no reported association with stones to date: rs61729229 in *VPS16 (vacuolar protein sorting 16 homolog*; OMIM 608550), rs201441165 in an isoform of *HAVCR1* (*Hepatitis A Virus Cellular Receptor 1*; *kidney injury molecule 1*, *KIM*‐*1*; OMIM 606518); rs15121559 in *PLA2R1 (phospholipase A2 receptor 1*; OMIM 604939), and rs140419507 in *PKP4* (*plakophilin 4*, *catenin 4*; OMIM 604276). The *VPS16* and *HAVCR1* variants segregated with stone formers and not with non‐stone formers while *PLA2R1*, *MAP3K5*, *PKP4*, and *SLC25A25* (Figure 1a; Figure 2) showed incomplete segregation and were initially assigned lower priority.

WES screening of II‐2 and III‐3 in the Italian kindred identified the same rs140777921 variant of *SLC25A25*, which was verified by Sanger sequencing. The variant segregated completely with stone formation (Figure 1b); and occurred in only three of the 12 non‐stone formers tested. The *VPS16* variant was not associated with stones in this kindred.

### 3.4 Comparisons with data from UK Biobank

Table 1 shows the frequencies of the rare variants of *SLC25A25*, *VPS16*, *PLA2R1*, *MAP3K5*, and *PKP4* in the UK Biobank data for UK stone formers and non‐stone formers. There were no statistically significant differences that withstood multiple testing correction between the allele frequencies in the two groups, suggesting that these rare variants are not associated with stone disease at the population level.

**TABLE 1:** Frequencies of five rare variant alleles from data held on the UK Biobank for DNA from 6,536 stone formers and 388,508 non‐stone formers

Gene	SNP	Locus^a^	Major allele	Minor allele	Stone formers		Non‐stone formers		Chi‐squared statistic	*p* value^b^
					Minor allele count	Minor allele frequency	Minor allele count	Minor allele frequency		
*SLC25A25*	rs140777921	chr9 130868670	G	C	29	0.002222	2534	0.003265	4.3180	.03772
*VPS16*	rs61729229	chr20 2846052	T	C	52	0.003985	3119	0.004021	0.0042	.9485
*PLA2R1*	rs151215519	chr2 160862183	T	G	26	0.00199	1633	0.002102	0.0770	.7814
*MAP3K5*	rs41288957	chr6 136882715	C	T	149	0.0117	8362	0.0110	0.5629	.4531
*PKP4*	rs140419507	chr2 140419507	C	T	21	0.001628	1467	0.001912	0.5385	.4630

Note

Population frequencies for the minor C allele of the *SLC25A25* variant from gnomAD v3.1.1: 0.0018.

^a^ dbSNP: GRCh37.p13 build.

^b^ A Bonferroni‐corrected *p* < .01 (0.05/5 tests) was set as the significance threshold.

### 3.5 APC3 isoforms: selection of APC3b

APC3 has four isoforms generated by alternative gene splicing, resulting in proteins with a common C‐terminal region but with variations in the N‐terminal region. Of these, mRNA for APC3a is widely expressed, highest in skeletal and heart muscle and pancreas, and moderate in the kidneys. mRNA for APC3b has a more restricted and lower expression, limited to kidneys, brain, and lungs. The amino acid sequences are identical from residue 54–469 (APC3a) and 87–503 (APC3b), and the main difference is that APC3a has three EF hands in the N‐terminal region, whereas APC3b has four (del Arco & Satrústegui, 2004; Satrústegui et al., 2007). The rs140777921 SNP is a G/C change in the *SLC25A25* gene; Chr 9: 130868670 G>C (GRCh37.P13 build). It is a missense variant which leads to substitution of histidine for glutamine in the transcripts of both the APC3a isoform (p.Gln315His in NM_052901.4) and the APC3b isoform (p.Gln349 His in NM_001006641.3).

It also overlaps four other *SLC25A25* transcripts not expressed in the kidneys. It is rare (MAF_gnomAD v3.1.1_ = 0.0018; MAF_Southampton Database_ = 0.0033), at a highly conserved site (Phylop: 0.999532; Gerp++: 5.08), and predicted to be deleterious by in silico analysis (Combined Annotation Dependent Depletion, CADD v1.6: 26; Rentzsch et al., 2021). Three other stone‐forming kindreds investigated by WES to date in the Southampton study do not have the variant. We opted to study APC3b because of the renal phenotype of the families and because this transcript was investigated previously in a study of TRPP2, a component of the renal polycystin 2 complex (Hofherr et al., 2018).

### 3.6 Modeling: The p.Gln349His variant could lead to destabilization of the domain structure of APC3b

**FIGURE 3:**
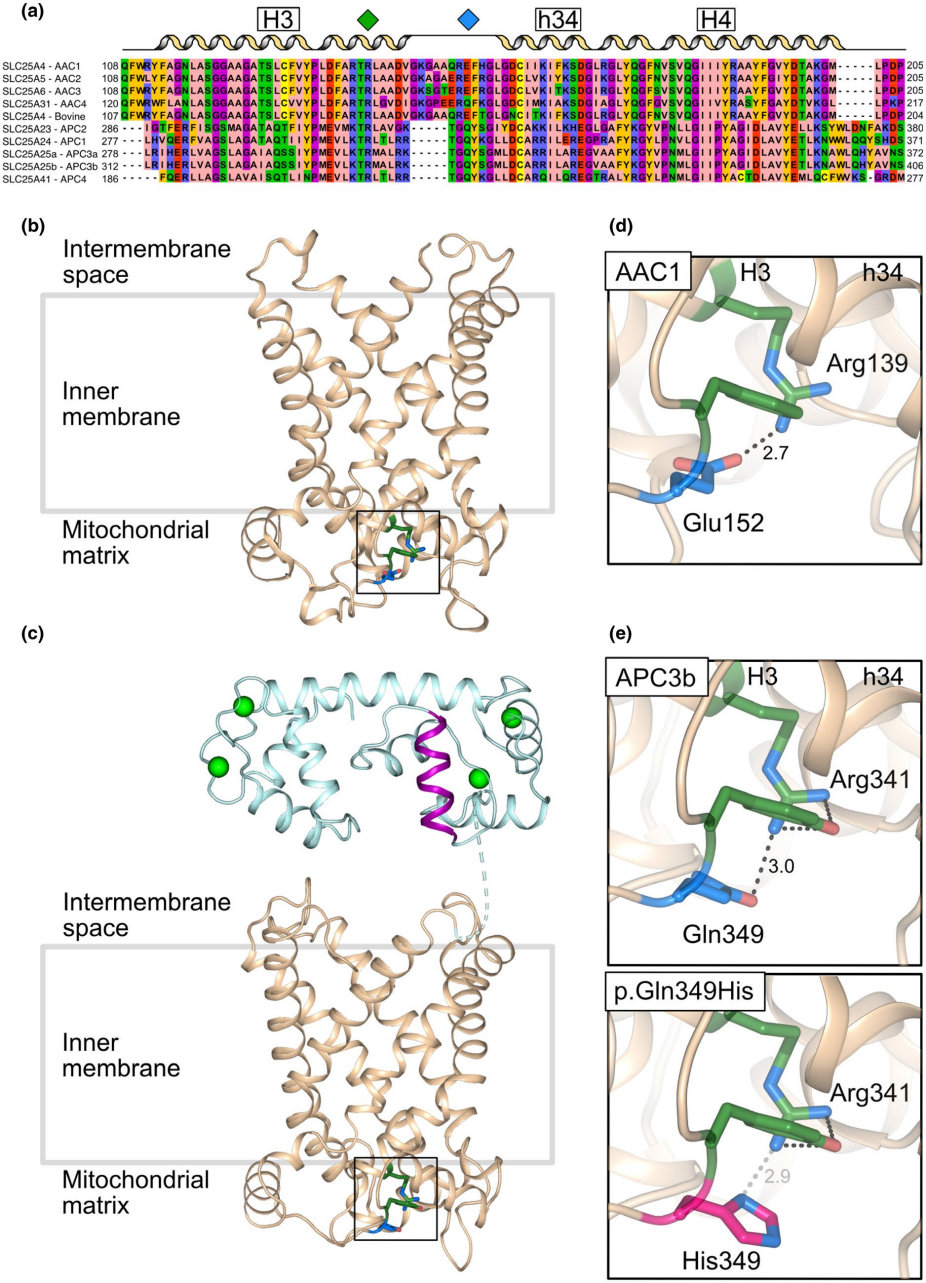
The glutamine to histidine substitution at position 349 in APC3b may compromise an intradomain interaction (a) Amino acid alignment of human ADP/ATP carrier paralogues 1–4 (AAC1‐4), bovine ADP/ATP carrier 1 (AAC1) and ATP‐Mg/Pi carrier paralogues (APC1, APC2, APC3a, APC3b, and APC4). The residues participating in the glutamine/glutamate‐arginine interaction in the interface between the odd number helix (H3) and the matrix helix (h34) in domain 2 are indicated with green (arginine) and blue (glutamine/glutamate) diamonds. Lateral view of (b) bovine AAC1 (PDB: 1okc) and (c) APC3b from the membrane in the cytoplasmic‐open state, showing the residues described in (a) The regulatory domain (cyan), amphipathic helix (purple), carrier domain (wheat), calcium ions (green spheres) are shown. Enlarged view of intradomain interactions of (d) AAC1 and (e) APC3b wild‐type (top) and p.Gln349His (bottom). The arginine (green), glutamine/glutamate (blue) and the pathogenic variant p.Gln349His (pink), the interactions (gray dash) and distances (Å) are indicated. The tyrosine (green) in APC3b (not present in AAC1) may also contribute to the stabilization of domain 2. The model of APC3b was generated using MODELLER (Webb & Sali, 2014) using the bovine AAC1 (PDB: 1okc) and the yeast ADP/ATP carrier structures (PDB: 4c9g, 4c9h, 4c9j, and 4c9q) as a template (Pebay‐Peyroula et al., 2003; Ruprecht et al., 2014)

APC3 alters the adenine nucleotide pool in the mitochondrial matrix in order to meet increased demand for cellular ATP and energy. It is activated by an increase in cytosolic calcium (Amigo et al., 2013; Aprille, 1993). The transporter has three structural domains; (a) a calmodulin‐like N‐terminal domain in the mitochondrial intermembrane space, which is involved in calcium regulation, (b) a loop domain with an amphipathic helix, and (c) a C‐terminal mitochondrial carrier domain with six transmembrane and three matrix helices, which is involved in transport of substrates (Harborne et al., 2015, 2017; Figure 3d). In the presence of calcium, the amphipathic helix is bound to the regulatory domain, allowing transport to occur (Harborne et al., 2015). In the calcium‐free state, the amphipathic helix is released, binds to the carrier domain and inhibits transport (Fiermonte et al., 2004; Harborne et al., 2017).

The variant is in the carrier domain, and hence could potentially affect the transport function of APC3b. The mutated Gln349 residue is conserved among all ATP‐Mg/Pi carriers and is in an equivalent position to a highly conserved glutamate residue in human ADP/ATP carriers (Glu152 in human adenine nucleotide translocase AAC1; Figure 3a). These residues interact with a positively charged amino acid located two residues downstream of a highly conserved Px[ED]xx[RK] motif of transmembrane helix 3 (H3; Miniero et al., 2011; Ruprecht et al., 2014), Arg139 in AAC1 and Arg341 in APC3b. A glutamine to histidine substitution introduces a bulkier and less flexible side chain (Figure 3e). There are fewer backbone‐dependent rotamers for the histidine side chain (3 out of 7) than for the glutamine side chain (8 out of 16), which could interact with Arg341. At the pH of the mitochondrial matrix (~pH 7.8), the imidazole ring of histidine is likely to be partially charged (pKa ~6), and depending on the position of the deprotonated nitrogen, p.Gln349His is either 2.9 Å or 4.6 Å distance from Arg341 (Li & Hong, 2011). The substitution could therefore disrupt the interaction with Arg341 and destabilize the domain. Further, Gln349 precedes [YF]xG, a highly conserved motif at the N‐terminus of the matrix helices involved in binding cardiolipin, which is important for mitochondrial carrier function (Klingenberg, 2009; Pebay‐Peyroula et al., 2003; Ruprecht et al., 2014, 2019; Figure S1). Disturbance of cardiolipin binding could be another destabilizing factor.

### 3.7 The transport activity of the p.Gln349His variant is severely affected

**FIGURE 4:**
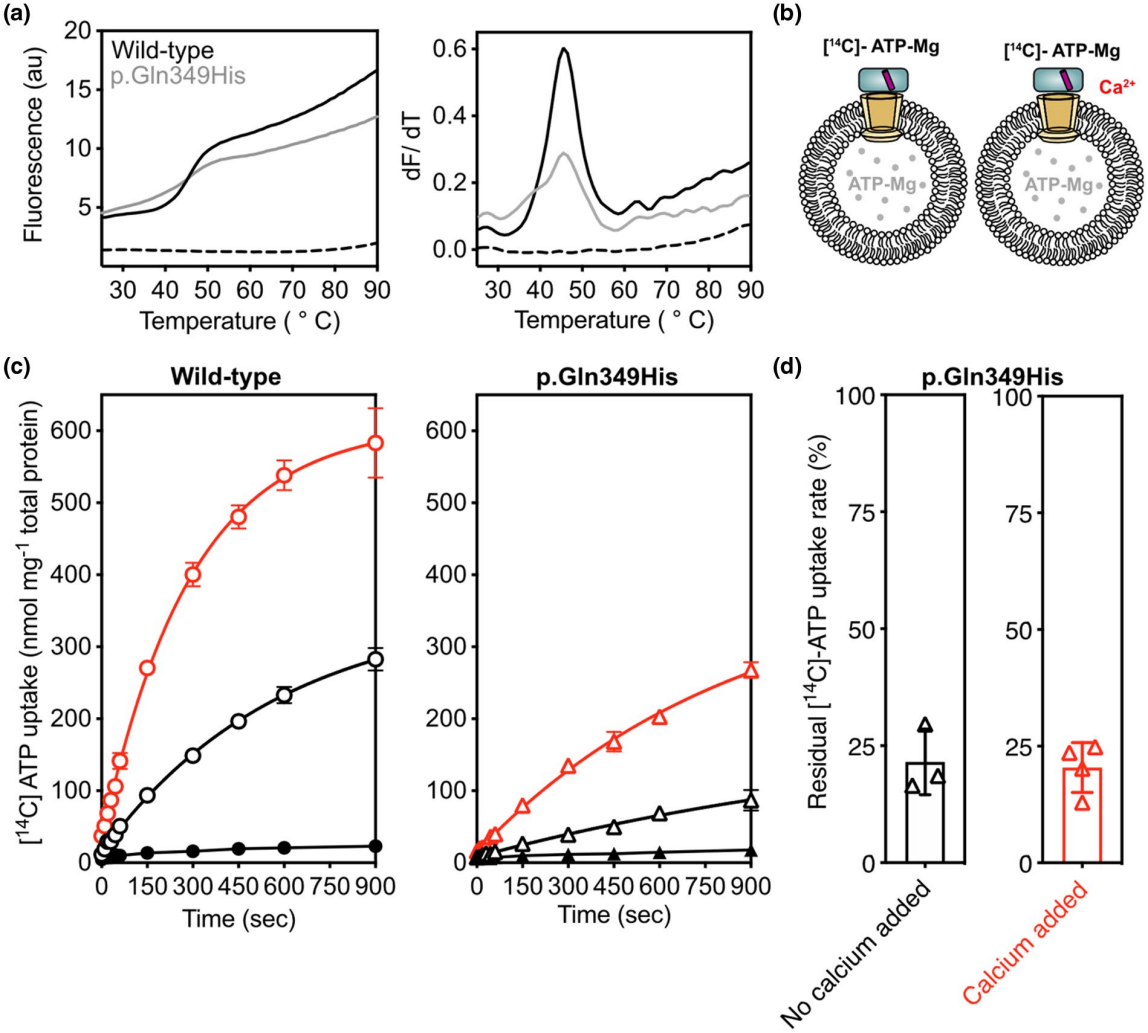
Effect of p.Gln349His on the thermal stability and transport activity of APC3b. (a) Thermostability profile (left), and its corresponding first derivative (right) of APC3b wildtype and p.Gln349His. (b) Schematic representation of proteoliposomes and the conditions tested. (c) A representative uptake curve showing the uptake of [^14^C]‐ATP‐Mg into proteoliposomes for APC3b wildtype (left) and pGln349His (right) with (red line) and without (black line) added calcium (1 mM). The error bars represent the standard deviation of four technical replicates. (d) Residual transport activity of APC3b p.Gln349His relative to APC3b wildtype, based on the initial transport rate and corrected for background binding with (red) and without (black) addition of 1 mM calcium. The error bars represent the standard deviation of four independent experiments, and the uptake curves are fitted with a one‐phase association curve

To determine the effect of the variant on the transport activity, human wild‐type and p.Gln349His APC3b were expressed in yeast mitochondria and purified. The protein purity and yield for the p.Gln349His variant were comparable to wild type, indicating that the variant did not impact the expression and targeting of the carrier (Figure S2). Thermal stability assays were used to test whether the p.Gln349His mutation had an effect on the apparent melting temperature (*T_m_*), a relative measure of protein stability (Figure 4a). Both wild type and p.Gln349His gave an apparent *T_m_* of 45.5℃ (three technical repeats) for the folded form in the presence of calcium. However, the variant displayed a higher baseline, a decreased derivative peak, and a series of folding intermediates at lower melting temperatures, indicative of a partially folded protein population. These observations agree with the notion that the protein itself is active, but that a part of the protein population might be unstable. Uptake assays were performed to assess the effect of the p.Gln349His variant on the transport activity. Purified wild type and p.Gln349His variant were pre‐treated with the calcium chelator EGTA (10 mM) in order to minimize free calcium in the sample, before being exchanged into assay buffer.

Wild‐type and p.Gln349His APC3b were reconstituted into proteoliposomes and the uptake of radio‐labelled ATP was monitored with and without calcium. In the presence of calcium, the uptake assays showed transport activity for both, but the overall activity of the p.Gln349His variant was only 21.6 ± 7% of the wild type (4 independent biological repeats). In the absence of calcium, transport by the p.Gln349His variant was similarly decreased to only 20.4 ± 5.3% residual activity (4 independent biological repeats; Figure 4). These results indicate that the p.Gln349His variant impacts the specific transport rate of ATP‐Mg, without affecting calcium regulation, which could be due to an unfolded subpopulation.

## 4 DISCUSSION

This study found that a rare autosomal dominant inherited missense variant of the gene for APC3, a mitochondrial ATP‐Mg transporter, is associated with renal calcium stones with incomplete penetrance. The mutated gene encodes a dysfunctional protein.

APC3 mediates the net import or export of adenine nucleotides in mitochondria. Since it has calcium binding EF‐hand motifs facing the extra‐mitochondrial space, the activity of the transporter is regulated by cytosolic calcium. Hence APC3 can transduce calcium signals into the mitochondria without requiring calcium entry into the matrix (del Arco & Satrústegui, 2004; Fiermonte et al., 2004). Structural modeling shows that the affected residue in the p.Gln349His variant is positioned in the interface between transmembrane helix 3 (H3) and the matrix helix (h34) interacting with Arg341, which might be important for stability of the transport protein. The transport activity of the p.Gln349His variant was significantly reduced to approximately one fifth of that of APC3b wild type, whereas the calcium regulation was unaffected. Thermostability assays show that a subfraction of the carrier population might be unfolded, which would explain the lower specific activity. The in vivo activity of the mutant carrier paired with a wild type allele in the biallelic state, as in the stone patients, cannot be predicted from these experiments. However, heterozygous missense mutations of the gene for APC1, *SLC25A24*, present with a severe developmental phenotype (Ehmke et al., 2017; Writzl et al., 2017), indicating that mutations of this closely related protein have a dominant negative effect.

To date there are no reports of APC3 deficiency in humans and few reports from experimental animal models. In a study to address the effects of APC3 deficiency on energy metabolic efficiency, mice with global *Slc25a25* deletion were viable at birth, had small decreases in both fat and lean body mass after weaning, reduced exercise endurance and were resistant to diet‐induced obesity. The kidneys were not examined and renal function was not investigated. Fibroblasts from *Slc25a25* ^−/−^ mouse embryos had decreased mitochondrial respiration and ATP, and decreased flux of Ca^2+^ across the endoplasmic reticulum (Anunciado‐Koza et al., 2011). There was no evidence of abnormally low exercise tolerance in the Southampton family. The statin‐induced muscle pain in one individual is of questionable relevance.

The *SLC25A25* disease variant, associated with stones, could impair the ability of the mitochondrion to regulate the production of ATP. Many primary mitochondrial disorders may affect the kidneys, with a wide range of damaging effects. Stones and/or nephrocalcinosis have been reported in patients with mitochondrial depletion, in which dNTP pools may be reduced, in Kearns Sayer Syndrome, and in non‐specific multiorgan mitochondrial disorders (Finsterer & Scorza, 2017).

The kidneys have a high energy requirement for transport of minerals and other components of the renal filtrate, with ATP consumption exceeding 2 kg/day (Walker, 2019). ATP depletion is likely to impact significantly on solute reabsorption throughout the nephron and this could lead to production of lithogenic urine. Metabolomic investigation of human *distal* renal tubular cells in vitro found that ATP was significantly reduced by *SLC25A25* knock‐down, and that concentrations of 42 metabolites changed significantly (Hofherr et al., 2018). The first tangible evidence for the importance of SLC25A25 as an energy supplier in the kidney in vivo is from a study investigating genome‐wide gene expression (mRNA) in response to acute and prolonged metabolic acidosis in proximal renal tubular cells from mice. *Slc25a25* was one of the genes with highest increase in expression in both conditions. Oxidative phosphorylation was the most upregulated cell pathway. It was proposed that in combination these responses supply ATP to fuel membrane transporter processes (Nowik et al., 2008).

Half of the Southampton family members, all with the variant, had hyperphosphaturia. This is common among stone formers, affecting around 30%. The explanation is unknown. Phosphate reabsorption occurs in the proximal tubules and is energy‐dependent. In this kindred the rare *SLC25A25* polymorphism might have contributed to hyperphosphaturia. Although hyperphosphaturia alone is unlikely to cause stones, it may do so in the presence of other urine abnormalities. The urinary profiles in 1998 did not include glucose, amino acids or low molecular weight proteins to investigate proximal tubular dysfunction specifically. However, there is tangential evidence that two functions of the proximal tubular functions were not significantly compromised at this time: normal citrate excretion indicates that the intracellular pH of the luminal epithelium was maintained within physiological limits, and excretion of oxalate was normal. This is regulated, in part, by proximal tubular carriers. Calcium was well absorbed and hypercalciuria was not a risk factor for stones in this family. No biochemical data are available for the Italian kindred.

As well as serving as an energy source, ATP is discharged extracellularly throughout the nephron for autocrine/paracrine purinergic signaling when renal cells are stretched by an increase in cell volume due to hypo‐osmolarity or to pressure changes in the renal tubules, or are stimulated by high flow rates through the tubular lumen bending cilia on the apical cell membranes. These stimuli trigger calcium release from intracellular stores, and ATP discharge through the plasma membrane, probably via pannexin channels (Burnstock et al., 2014; Praetorius & Leipziger, 2013). The intracellular source for this burst of ATP for purinergic signaling is unknown. In polymorphonuclear neutrophils, it was shown to be the mitochondria (Bao et al., 2014). Recent studies by Hofherr et al., (2018) implicate APC3 in the cilia‐triggered response to flow. They demonstrated that APC3 is one of the calcium‐activated proteins that acts downstream of the polycystin‐2 (TRPP2) ion channel, a component of the polycystin complex in cilia. In addition, knock‐down of *Slc25a25* in zebra fish larvae disrupted normal body lateralization, typical of cilia dysfunction. In the distal renal tubules, purinergic stimulation normally protects the body from fluid overload by reducing sodium and water reabsorption, promoting a diuresis of dilute urine (Burnstock et al., 2014; Praetorius & Leipziger, 2013). A defective purinergic response might increase the concentration of minerals at this site and the risk of crystallization. To date, this possibility has received scant attention. The capacity to dilute urine was not tested in the Southampton family. Bile flow is similarly stimulated by flow and hypotonicity, calcium‐induced ATP release and purinergic signaling (Gradilone et al., 2007). It is possible that this response was reduced by the variant, contributing to gallstone formation in this kindred. Gallstone and kidney stone formation are independently associated (Taylor et al., 2011). Further studies in an animal model are required to explore the effects of the *SLC25A25* variant in vivo.

In the families reported, the *SLC25A25* mutation was not a fully penetrant cause of stone formation, indicating that in isolation it has only a modest effect on the susceptibility to stones. Other gene variants are likely to act as important modifiers to compound the risk, as in other complex polygenic disorders (Palsson et al., 2019; Pang et al., 2021). Possibilities in the Southampton family are those identified in *VPS16* and *HAVCR1* which code for a cell trafficking protein and the kidney injury molecule, KIM1, respectively, and the three considered less likely because of poor segregation with stones: *PLA2R1*, *MAP3K5*, and *PKP4*. Five large cross‐sectional population GWAS studies have looked for associations between stones and common gene variants, two from Iceland (Oddsson et al., 2015; Thorleifsson et al., 2009), two from Japan (Tanikawa et al., 2019; Urabe et al., 2012) and one from the UK (Howles et al., 2019). The majority of the variants detected are non‐coding, and functional studies to assess their relevance are lacking. It is notable that only six of the reported genes mapped closely to genes for membrane channels or transporters. The roles of many of the others in renal function or mineral turnover are currently unclear.

The Analysis of data from the UK Biobank data set did not demonstrate a statistically significant association of the *SLC25A25*, *VPS16*, *PLA2R1*, *MAP3K5*, and *PKP4* variants with stones. This can be explained by lack of statistical power for rare variant detection. Unless the polymorphism has a large effect on phenotype, extremely large study populations are required to demonstrate statistical associations for rare variants. In addition, due to the wide phenotypic heterogeneity of calcium stone disease, inaccurate reporting may have led to misclassification of the Biobank study participants into stone formers/non‐stone formers. This would impact significantly on the size of the small group with the variant, whilst having little effect on the large group with the common allele (Palsson et al., 2019; Pang et al., 2021).

This is the first report which links stones to a dysfunctional mitochondrial transporter. The exciting feature of this gene is that it is regulated by changes in intracellular calcium which, in turn, mediate cell signaling and ATP release. APC3 may be a key intermediary for adapting mitochondrial activity to molecular transport at the cell surface. Applied selectively to families or cohorts of individuals with a clearly defined clinical and biochemical phenotype, WES has great capability to identify rare variants that contribute to stone risk which could not be predicted from our current limited understanding. It is essential that novel, unexpected associations, are explored by functional studies. Data and experience must be shared to progress our understanding of recurrent stone formation and prevention strategies.

## ETHICAL COMPLIANCE

All the studies were conducted according to the Helsinki Declaration with ethical committee approval. Written and informed consent was obtained from all participants. The first Southampton Study was approved by the Southampton and South West Hampshire Research Ethics Committee (1997; Ref 322/970) and the second by the East of England‐ Cambridge Central Research Ethics Committee (2016; Ref 16/EE/0293). The Italian study was performed according to the Italian National Bioethics Committee. UK Biobank has approval from the North West Multi‐Centre Research Ethics Committee (11/NW/0382). The reported study (Epidemiology of Kidney Stone Disease) has UK Biobank Study ID 885.

## CONFLICT OF INTEREST

M.R. Jabalameli, F. Fitzpatrick, R. Colombo, S.A. Howles, E.R.S. Kunji, G. P. Leggatt, V. Walker, A. Wiberg, and S. Ennis, declare that they have no conflict of interest.

## AUTHOR CONTRIBUTIONS

MRJ, GL, and SE undertook and interpreted the genomic studies in Southampton. RC investigated the Italian pedigree and coordinated genomic studies in his laboratory. ERSK undertook computational modeling of SLC25A25 and the disease variant, and FF and ERSK carried out the functional studies. SH coordinated the Biobank study and SH and AK analyzed the Biobank data. VW conceived and coordinated the Southampton study and drafted the manuscript. All Authors contributed to the final manuscript.

## Supporting information

Fig S1

Fig S2

Table S1

Table S2

Table S3

Document S1

## Data Availability

The data that supports the findings of this study are available in the supplementary material of this article.
