# Supplementary material for: Exome sequencing identifies a disease variant of the mitochondrial ATP‐Mg/Pi carrier SLC25A25 in two families with kidney stones
Source: Mol Genet Genomic Med. 2021 Aug 4;9(12):e1749. doi: 10.1002/mgg3.1749 (PMC8683635; doi:10.1002/mgg3.1749)
Supplement: Supplementary file 1 — Fig S1 [file MGG3-9-e1749-s001.docx]

**Figure S1 Cardiolipin binding in APC3b**

1. APC3b modelled with three cardiolipin molecules (ball-and-stick representation) found in the crystal structure of AAC (PDB: 2c3e) (Nury *et al* 2005). The cardiolipin acyl chains are partially modelled.
2. An enlarged view of cardiolipin-binding to the positively charged ends of the helix dipoles in the even-numbered (H2) and matrix (h34) helices.

The dipoles are colored using a gradient electrostatic potential (blue (positive) to red (negative). The residues in the conserved cardiolipin-binding motif [YWF][RK]G (purple) and [YF]xG (green) are represented by sticks. Hydrogen and electrostatic interactions are shown by dashed lines (cyan). Glutamine 349 (blue stick representation) in APC3b is preceding [YF]xG, a highly conserved cardiolipin binding motif.

Nury H, Dahout-Gonzalez C, Trézéguet V, Lauquin G, Brandolin G, Pebay-Peyroula E. Structural basis for lipid-mediated interactions between mitochondrial ADP/ATP carrier monomers. *FEBS Lett* 2005; **579**: 6031–6036
