# Supplementary material for: Exome sequencing identifies a disease variant of the mitochondrial ATP‐Mg/Pi carrier SLC25A25 in two families with kidney stones
Source: Mol Genet Genomic Med. 2021 Aug 4;9(12):e1749. doi: 10.1002/mgg3.1749 (PMC8683635; doi:10.1002/mgg3.1749)
Supplement: Supplementary file 2 — Fig S2 [file MGG3-9-e1749-s003.docx]

**Figure S2 Purification of APC3b wild-type and p.Gln349His**.

Purified protein (5 µg) in 3:1 mix with loading buffer was separated using precast 4-12 % TruPAGE^™^ SDS-PAGE gels and stained using Instant*Blue*^™^ Coomassie.
