# Supplementary material for: Exome sequencing identifies a disease variant of the mitochondrial ATP‐Mg/Pi carrier SLC25A25 in two families with kidney stones
Source: Mol Genet Genomic Med. 2021 Aug 4;9(12):e1749. doi: 10.1002/mgg3.1749 (PMC8683635; doi:10.1002/mgg3.1749)
Supplement: Supplementary file 3 — Table S1 [file MGG3-9-e1749-s005.docx]

**Table S1 : The panel of candidate genes used in the first round of analysis of the exome sequence data from three stone formers in the Southampton kindred.**

The list of genes (n=366) was compiled from a web search of publications up to 2016 of papers which related to stones, renal function and absorption, metabolism, turnover and excretion of minerals and organic stone constituents.

| ABCC4 |  | ATP6V1G1 | | EDN2 |  | GRK6 |  | NR3C1 |  | REN |  | SLC22A2 |  | TNSF11 |
| --- | --- | --- | --- | --- | --- | --- | --- | --- | --- | --- | --- | --- | --- | --- |
| ABCG2 |  | ATP6V1G2 | | EDN3 |  | HAS1 |  | NR3C2 |  | RGS10 |  | SLC22A6 |  | TPCN1 |
| ADCY10 |  | ATP6VIG3 | | EDNRA |  | HAS2 |  | NR4A2 |  | RGS12 |  | SLC22A7 |  | TRPM6 |
| ADCY5 |  | AVPR1A |  | EDNRB |  | HAS3 |  | NUDT19 |  | RGS14 |  | SLC22A7 |  | TRPM7 |
| ADCY6 |  | AVPR2 |  | EDNRB |  | HNF1A |  | OAT4 |  | RGS2 |  | SLC22A8 |  | TRPV5 |
| ADD1 |  | BSG |  | EGR1 |  | HNF1B |  | OCRL1 |  | RGS4 |  | SLC25A24 |  | TRPV6 |
| ADD3 |  | BSND |  | EMB |  | HPRT1 |  | PAK1 |  | RGS7 |  | SLC25A25 |  | TUBB |
| ADRA1A |  | CA12 |  | ENPP1 |  | HSP90 |  | PDE7B |  | RHBG |  | SLC26A1 |  | TYK2 |
| ADRA1B |  | CA2 |  | ENPP3 |  | HSP90AA1 | | PDZD3 |  | RHCG |  | SLC26A2 |  | UMOD |
| ADRA1D |  | CA4 |  | EZR |  | HSP90AB1 | | PDZK1 |  | RHOA |  | SLC26A26 |  | URAT1 |
| ADRA2A |  | CALB1 |  | F12 |  | INMT |  | PFN3 |  | RIPK2 |  | SLC26A3 |  | VDR |
| ADRA2B |  | CALM1 |  | F2 |  | IP6K3 |  | PHEX1 |  | RNLS |  | SLC26A7 |  | XDH |
| ADRA2C |  | CALM2 |  | FAM188B |  | ITGB1 |  | PICK1 |  | ROCK1 |  | SLC26A9 |  | YWHAB |
| ADRB1 |  | CALM3 |  | FGF23 |  | ITIH1 |  | PIK3C2A |  | S100A12 |  | SLC2A9 |  | YWHAE |
| ADRB2 |  | CAMK1 |  | FGF6 |  | ITIH2 |  | PIK3C2B |  | S100A6 |  | SLC34A1 |  | YWHAZ |
| ADRB3 |  | CAMK2 |  | FGF7 |  | ITIH3 |  | PIK3CA |  | S100A8 |  | SLC34A2 |  | ZNF365 |
| ADRBK1 |  | CASR |  | FGFR1 |  | JAK1 |  | PIK3CB |  | S100A9 |  | SLC34A3 |  |  |
| ADRBK2 |  | CAV1 |  | FGFR3 |  | JAK2 |  | PKD1 |  | S100G |  | SLC3A1 |  |  |
| AGTR1 |  | CCDC88A |  | FGFR4 |  | JAK3 |  | PKD2 |  | SCGN |  | SLC41A1 |  |  |
| AGTR2 |  | CCL2 |  | FLNA |  | KCNH2 |  | PKHD1 |  | SCNN1A |  | SLC47A1 |  |  |
| AGXT |  | CD38 |  | FN1 |  | KCNJ1 |  | PLCG1 |  | SCNN1B |  | SLC47A2 |  |  |
| AKAP12 |  | CD44 |  | FXYD2 |  | KCNK5 |  | PMCA1b |  | SCNN1G |  | SLC4A1 |  |  |
| AKAP5 |  | CD44 |  | FXYD2 |  | KIAA0474 |  | PMCA4b |  | SFRP4 |  | SLC4A2 |  |  |
| ALPL |  | CHP1 |  | FXYD5 |  | KL |  | PP3CC |  | SGK1 |  | SLC4A3 |  |  |
| AMBP |  | CICA1 |  | GALNT3 |  | LRP2 |  | PPP1R16B | | SHC1 |  | SLC4A4 |  |  |
| ANKH |  | CICA2 |  | GALNT3 |  | LRRC16A |  | PPP1R1B |  | SHROOM3 | | SLC4A7 |  |  |
| ANXA2 |  | CLCN1 |  | GBSM2 |  | MAP3K14 |  | PPP3CA |  | SLC12A1 |  | SLC5A1 |  |  |
| ANXA3 |  | CLCN5 |  | GCKR |  | MAP3K5 |  | PPP3CB |  | SLC12A3 |  | SLC5A12 |  |  |
| AQP1 |  | CLCNKA |  | GHRPR |  | MAPK1 |  | PPP3R1 |  | SLC13A2 |  | SLC5A8 |  |  |
| AQP2 |  | CLCNKB |  | GNA11 |  | MAPK10 |  | PPP3R2 |  | SLC13A2 |  | SLC7A9 |  |  |
| AQP3 |  | CLD19 |  | GNA12 |  | MAPK2 |  | PRKACA |  | SLC13A3 |  | SLC8A1 |  |  |
| AQP4 |  | CLDN14 |  | GNA13 |  | MAPK3 |  | PRKCA |  | SLC13A3 |  | SLC9A1 |  |  |
| ARRB1 |  | CLDN16 |  | GNAI1 |  | MAPK7 |  | PRKCB |  | SLC13A4 |  | SLC9A2 |  |  |
| ARRB2 |  | CLU |  | GNAO1 |  | MEN1 |  | PRKCD |  | SLC13A4 |  | SLC9A3 |  |  |
| ATP12A |  | CRAF |  | GNAQ |  | MEPE |  | PRKCE |  | SLC13A5 |  | SLC9A3R1 |  |  |
| ATP1A1 |  | CYP19A1 |  | GNAS |  | MGP |  | PRKCG |  | SLC16A1 |  | SLC9A3R2 |  |  |
| ATP1B1 |  | CYP19A1 |  | GNAS1 |  | MMP2 |  | PRKCZ |  | SLC16A3 |  | SLC9A4 |  |  |
| ATP2B1 |  | CYP24A1 |  | GNB1 |  | MTNR1A |  | PRPS2 |  | SLC16A4 |  | SLCO4A1 |  |  |
| ATP2B3 |  | CYP27B1 |  | GNB2 |  | MYO6 |  | PTH1R |  | SLC16A7 |  | SNX27 |  |  |
| ATP4A |  | DDC |  | GNB2L1 |  | NADSYN1 |  | PTK2 |  | SLC16A9 |  | SOD2 |  |  |
| ATP4B |  | DGKH |  | GNB4 |  | NBPF3 |  | PTPN11 |  | SLC17A1 |  | SOD3 |  |  |
| ATP6V0A1 | | DMP1 |  | GNB5 |  | NOS1 |  | PTPRE |  | SLC17A2 |  | SOS1 |  |  |
| ATP6V0A2 | | DPP4 |  | GNG12 |  | NOS2 |  | PYK2 |  | SLC17A3 |  | SPP1 |  |  |
| ATP6V0A4 | | DRD1 |  | GNG13 |  | NOS3 |  | RAD51 |  | SLC17A4 |  | SPP1 |  |  |
| ATP6V0D2 | | DRD2 |  | GNG2 |  | NPPA |  | RAD51AP1 | | SLC20A1 |  | SRC |  |  |
| ATP6V1B1 | | DRD3 |  | GNG5 |  | NPPB |  | RAD51AP1 | | SLC20A2 |  | STAT1 |  |  |
| ATP6V1B1 | | DRD4 |  | GPC3 |  | NPPC |  | RAP1GAP |  | SLC22A11 |  | STC1 |  |  |
| ATP6V1B2 | | DRD5 |  | GPSM1 |  | NPR1 |  | RAPGEF3 |  | SLC22A12 |  | TCIRG1 |  |  |
| ATP6V1C2 | | ECE1 |  | GRB2 |  | NPR2 |  | RDX |  | SLC22A13 |  | TFF1 |  |  |
| ATP6V1E2 | | EDN1 |  | GRK5 |  | NPR3 |  | REG1A |  | SLC22A18 |  | TNFSF11 |  |  |
