# Supplementary material for: Exome sequencing identifies a disease variant of the mitochondrial ATP‐Mg/Pi carrier SLC25A25 in two families with kidney stones
Source: Mol Genet Genomic Med. 2021 Aug 4;9(12):e1749. doi: 10.1002/mgg3.1749 (PMC8683635; doi:10.1002/mgg3.1749)
Supplement: Supplementary file 4 — Table S2 [file MGG3-9-e1749-s006.docx]

**Table S2 major illnesses recorded for the Southampton family from their questionnaires (2016) and Hospital records (2020)**

| Family member | illnesses |
| --- | --- |
| Stone formers |  |
| [I_4] 3 | [deceased prior to 1998; nephrectomy for stones; gall stones] |
| [II_5 19 | [deceased prior to 1998; stone at 64y; no details] |
| [II_8] 21 | [deceased prior to 1998; stone at 43y; myocardial infarction] |
| II_1 1 | Ischaemic heart disease; congestive cardiac failure; severe spinal osteoporosis noted at 81y; died 2007 (88y) |
| II_6 20 | Parkinsons disease; indwelling catheter 5 years; cerebobrovascular accident and died 1999 (87y) |
| III_5 23 | Hypertension, abdominal aortic aneurysm, myocardial infarction (aged late 50s); asthma; gallstones; died 2016 (86y) |
| III_7 30 | Hypertension; type II diabetes mellitus |
| Non-stone formers |  |
| [II-3 ] 18 | [deceased prior to 1998; myocardial infarction; gall stones] |
| III_1 36 | Hypertension; osteoarthritis; glaucoma |
| III_3 37 | Hypertension; arthritis |
| III_8 31 | No details |
| III_10 49 | No details |
| IV_1 39 | None serious |
| IV_2 40 | Palatoplasty; sleep apnoea |
| IV_3 42 | No details |
| IV_4 43 | None serious |
| IV_5 26 | Polygenic hypercholesterolaemia; muscle pains on statins |
| IV_6 27 | None serious; discectomy for spinal injury |
| IV_7 28 | Hypertension; Méniѐres disease; |
| IV_8 34 | No details |
| IV_9 33 | No details |
