# Supplementary material for: Exome sequencing identifies a disease variant of the mitochondrial ATP‐Mg/Pi carrier SLC25A25 in two families with kidney stones
Source: Mol Genet Genomic Med. 2021 Aug 4;9(12):e1749. doi: 10.1002/mgg3.1749 (PMC8683635; doi:10.1002/mgg3.1749)
Supplement: Supplementary file 5 — Table S3 [file MGG3-9-e1749-s002.docx]

**Table S3 :** **Biochemistry of members of family 1 in 1998 whose DNA in stored blood was analysed in the follow-up study twenty years later.**

| Family member | II-1^a^ | II-6 | III-5^a^ | III-7^a^ | *III-1* | *III-10* | IV-1 | IV-2 | IV-4 | IV-5 | IV-6 | IV-7 |
| --- | --- | --- | --- | --- | --- | --- | --- | --- | --- | --- | --- | --- |
| Sex | F | M | M | M | M | M | F | M | F | F | M | F |
| Age (y) in 1998 | 79 | 86 | 67 | 49 | 57 | 43 | 31 | 29 | 32 | 39 | 36 | 29 |
| Age (y) when stones | 44, 78 | 85y ⴕ | 38 | 18, 28 |  |  |  |  |  |  |  |  |
| ***24h urine*** |  |  |  |  |  |  |  |  |  |  |  |  |
| calcium (mmol/L) | 2.02 | 2.99 | 0.99 | 4.49 | 4.84 | 2.44 | 3.97 | 3.34 | 2.33 | 2.22 | 7.31 | 2.41 |
| calcium (mmol/24h) | 4.22 | 4.31 | 1.65 L | 6.87 | 5.32 | 3.93 | 4.33 | 4.64 | 4.45 | 4.44 | 9.36 H | 3.59 |
| calcium reabsorbed/24h % | 97.11 | 95.3 | 98.98 | 97.58 | 97.34 | 98.48 | 98.04 | 98.19 | 98.15 | 97.71 | 96.82 | 98.36 |
| oxalate (mmol/24h) | 0.31 | 0.42 | 0.27 | 0.34 | 0.44 | 0.45 | 0.28 | 0.56 H | 0.34 | 0.28 | 0.29 | 0.49 |
| citrate (mmol/24h) | 2.15 | 1.09 L | 3.47 | 3.76 | 2.64 | 3.72 | 5.33 | 3.79 | 5.33 | 5.74 | 7.16 | 3.93 |
| magnesium (mmol/24h) | 2.51 | 4.32 | 3.67 | 4.28 | 3.41 | 6.12 | 4.25 | 5.56 | 4.39 | 3.80 | 8.32 H | 4.17 |
| volume (L/24h) | 2.09 | 1.44 | 1.67 | 1.53 | 1.10 | 1.61 | 1.09 | 1.39 | 1.91 | 2.00 | 1.28 | 1.49 |
| creatinine (mmol/L) | 3.1 | 6.3 | 7.1 | 10.8 | 11.9 | 9.7 | 10.4 | 11.0 | 5.0 | 4.8 | 14.2 | 8.0 |
| creatinine clearance (L/24h) | 93.9 | 75.0 L | 117.4 | 194.4 | 148.8 | 190.5 | 157.4 | 186.5 | 167.5 | 139.1 | 202.0 H | 154.8 |
| ***Fasting random urine*** |  |  |  |  |  |  |  |  |  |  |  |  |
| calcium/creatinine (mmol/mmol) | 0.53 | 0.42 | 0.12 | 0.48 | 0.12 | 0.18 | 0.16 | 0.26 | 0.34 | 0.44 | 0.43 | 0.16 |
| tubular reabsorbed calcium% | 97.64 | 96.44 | 99.15 | 97.23 | 99.22 | 98.92 | 99.20 | 98.44 | 98.67 | 97.81 | 97.34 | 99.16 |
| citrate/creatinine (mmol/mmol) | 0.33 | 0.10 L | 0.24 | 0.23 | 0.14 L | 0.19 | 0.35 | 0.16 | 0.37 | 0.67 | 0.34 | 0.19 |
| creatinine (mmol/L) | 4.6 | 7.2 | 6.0 | 8.1 | 20.4 | 8.8 | 15.8 | 17.9 | 3.7 | 14.2 | 19.9 | 13.6 |
| pH | 6.8 | 6.2 | / | 6.2 | / | 5.0 | / | / | / | / | 5.2 | 5.0 |
| TmPO4/GFR (mmol/L) | 0.70 L | 0.60 L | 0.64 L | 0.78 L | 0.80 | 0.91 | 0.95 | 1.08 | 1.21 | 0.82 | 0.72 L | 0.48 L |
| ***Fasting blood*** |  |  |  |  |  |  |  |  |  |  |  |  |
| calcium (mmol/L) | 2.59 H | 2.35 | 2.31 | 2.43 | 2.24 | 2.26 | 2.34 | 2.29 | 2.39 | 2.32 | 2.43 | 2.36 |
| ultrafilterable calcium (mmol/L)-estimate (mmol/L) | 1.55 H | 1.41 | 1.39 | 1.46 | 1.34 | 1.36 | 1.40 | 1.37 | 1.43 | 1.39 | 1.46 | 1.42 |
| magnesium (mmol/L) | 0.80 | 0.82 | 0.77 | 0.93 | 0.82 | 0.74 | 0.84 | 0.76 | 0.86 | 0.81 | 0.82 | 0.78 |
| 1,25 vit D (pmol/L) | 70 | / | 67 | 145 H | 111 H | 56 | 107 | 63 | 94 | 107 | 93 | 102 |
| PTH ^b^ (pmol/L) | 2.0 | 10.6 H | 3.2 | 7.5 H | 5.9 | 1.9 | 2.7 | 3.8 | 1.8 | 1.2 | 2.6 | 2.2 |
| phosphate (mmol/L) | 0.87 | 0.94 | 0.90 | 1.03 | 1.01 | 1.10 | 1.14 | 1.30 | 1.14 | 0.91 | 1.02 | 0.71 |
| bicarbonate (mmol/L) | 31 | 27 | 29 | 26 | 25 | 28 | 26 | 29 | 29 | 28 | 29 | 26 |
| creatinine (μmol/L) | 69 | 121 H | 101 | 85 | 88 | 82 | 72 | 82 | 57 | 69 | 90 | 77 |
| urate (mmol/L) | 0.22 | 0.31 | 0.20 | 0.32 | 0.29 | 0.23 | 0.09 L | 0.26 | 0.13 | 0.11 L | 0.24 | 0.20 |
| ***SLC25A25 alleles*** |  |  |  |  |  |  |  |  |  |  |  |  |
| G1047 (ancestral); C1047 (rare) [protein transcript NM_001006641.3] | G/C | G/C | G/C | G/C | G/C | G/C | G/C | G/C | G/G | G/C | G/C | G/C |

Blue columns= stone formers (SFs). Results which are outside the reference ranges are highlighted in grey -H indicates high, & L low values. A low TmPO4/GFR value indicates phosphaturia

^a^ DNA from stone formers II-1, III-5 AND III-7 was analysed by exome sequencing. DNA from all 12 individuals was analysed for the SLC25A25 mutation G1047C (p Q349H) using Kaspar sequencing (KAP^TM^ ; LCG).

ⴕ stone was diagnosed at 85 years when symptomatic

^b^PTH = parathyroid hormone;

***Ref. Ranges*:** *Plasma*: calcium 2.15-2.55 mmol/L; estimated ultrafilterable plasma Ca (Ref 1.29-1.53 mmol/L; phosphate 0.70-1.50 mmol/L; bicarbonate 24-31 mmol/L; magnesium 0.74-1.03 mmol/L; PTH <7.3 pmol/L; 1,25 OH vitamin D 48-110 pmol/L; urate: men 0.15-0.45 mmol/L; women 0.12-0.36 mmol/L; creatinine men 80-115 μmol/L; women 53-97 μmol/L

*24H urine:* calcium: men 2.00-7.50 mmol/324h; women 2.00-6.25 mmol/24h;magnesium 1.7-6.8 mmol/24h; oxalate ≤ 0.50 mmol/24h; citrate ≥ 1.60 mmol/24h; creatinine clearance men 135-200 L/24h; women 120-180 L/24h

*Random urine* (adults): calcium ≤ 0.59 mmol/mmol creat; citrate ≥ 0.16 mmol/mmol creat; TmPO_4_/GFR 0.80-1.35 mmol/L (low values indicate phosphaturia)
