# Supplementary material for: Exome sequencing identifies a disease variant of the mitochondrial ATP‐Mg/Pi carrier SLC25A25 in two families with kidney stones
Source: Mol Genet Genomic Med. 2021 Aug 4;9(12):e1749. doi: 10.1002/mgg3.1749 (PMC8683635; doi:10.1002/mgg3.1749)
Supplement: Supplementary file 6 — Document S1 [file MGG3-9-e1749-s004.docx]

**Document S1 Questionnaire issued to family members in 2016 at the time of consenting to the follow up, genomics, study in 2016**

**Variations in our genes that increase the risk of having kidney stones**

**Please tick yes, no, or not applicable (N/A) as appropriate, provide the information requested in boxes 6 and 7 if it applies to you and write your name on the bottom of the sheet. Please return the completed form with your signed consent form to Dr Cook in the enclosed envelope**

|  | Yes | No | N/A |
| --- | --- | --- | --- |
| 1. Have you ever made a kidney stone? |  |  |  |
| 2. Have you made a kidney stone since the study in 1998-2000? |  |  |  |
| 3. Have any of your children made a stone? |  |  |  |
| 4. Have any of your grandchildren made a stone? |  |  |  |
| 5. Have any of your great grandchildren made a stone? |  |  |  |
| 6. Are you under a doctor for a chronic disorder such as diabetes, high blood pressure, angina, bone or joint problems?  If ‘yes’ please list them here |  |  |  |
| 7. Do you take any medications?  If ‘yes’ please list them here (doses not needed) |  |  |  |
| 8. Do you have children or grandchildren now aged 18 years or older who were too young to take part in the first study?  If ‘yes’ and you think that they might wish to take part in the new study please would you ask them to contact Dr Cook by email ([paul.cook@uhs.nhs.uk](mailto:paul.cook@uhs.nhs.uk)) and he will send them information |  |  |  |

Name:
